# On a Keller-Segel type equation to model Brain Microvascular Endothelial Cells growth’s patterns

**Published:** 2026-05-01

**Authors:** B. Ambrosio, A.F. Garroudji, S. Fitzsimons, H. Zaag, F.M. Elahi

**Affiliations:** a University Le Havre Normandie, Normandie Univ., LMAH UR 3821, Le Havre, 76600, France; b The Hudson School of Mathematics, 244 Fifth Avenue, Suite Q224, New York, 10001, NY, USA; c University Sorbonne Paris Nord, LAGA, CNRS (UMR 7539), Villetaneuse, 93430, France; d School of Medicine, Conway Institute, University College of Dublin, Belfield, Dublin, 4, Ireland; e Departments of Neurology and Neuroscience, Ronald M. Loeb Center for Alzheimer’s Disease, Friedman Brain Institute, Glickenhaus Center for Successful Aging, Icahn School of Medicine at Mount Sinai, 1 Gustave L. Levy Place, New York, 10029-5674, NY, USA

**Keywords:** Brain Microvasculature, Mathematical Modeling, Mathematical Analysis, Keller-Segel, Computational Neuroscience

## Abstract

This article presents a partial differential equation (PDE) of Keller–Segel (KS) type that reproduces patterns commonly observed during the growth of brain microvasculature. We provide mathematical insights into the mechanisms underlying the emergence of these patterns. In addition, we derive a data-driven equation that ensures a consistent temporal evolution of the chemoattractant associated with the observed microvascular dynamics. Beyond numerical simulations, the aim of this study is to advance a comprehensive mathematical modeling framework, spanning blood flow in cerebral arterial networks to biochemical processes, in order to better understand how vascular impairments may contribute to neurodegenerative diseases.

## 1. Introduction

Neurodegenerative diseases (ND) constitute a major and growing global health burden. It is estimated that more than 3 billion people currently live with neurological conditions [1], while dementia alone is projected to affect approximately 152 million individuals by 2050 [2]. Increasing evidence indicates that dysregulation of cerebral blood flow plays an important role in the development and progression of several forms of dementia [3, 4, 5, 6]. Consequently, understanding the structure and dynamics of the brain microvasculature in both healthy and pathological conditions is of central importance.

Recent advances in stem-cell technologies provide powerful tools to investigate the mechanisms of brain microvascular growth. In particular, induced pluripotent stem cells (iPSCs) can be differentiated into brain microvascular endothelial cells (BMECs). BMECs can also be directly isolated from the human brain, enabling the construction of multicellular *in vitro* models of the neuro–glio–vascular unit. These models offer a valuable framework for identifying the molecular drivers of pathological cell–cell interactions within this system [7].

The formation of vascular networks *in vitro* has been observed and studied for several decades (see, for example, [8]). More recently, the increasing availability of large-scale biological datasets and the development of computational analysis tools have created new opportunities to identify molecular markers associated with vascular pattern formation and to investigate correlations between spatial cellular patterns and omics data in the brain.

From the perspective of applied mathematics, pattern formation has long been studied through the framework of reaction–diffusion (RD) systems. These models are known to generate a wide variety of spatial and spatio-temporal structures through mechanisms such as Turing instabilities, Hopf bifurcations, phase-locked oscillations, and chaotic dynamics; see, for example, [9, 10, 11, 12]. In the context of cellular aggregation and pattern formation, a classical model is the Keller–Segel (KS) system [13], which incorporates both diffusive transport and chemotactic cell migration.

In this work, we study a Keller–Segel-type model capable of reproducing spatial patterns similar to those observed in BMEC cultures *in vitro*. We first present the model and provide a preliminary analysis of the associated ordinary differential equation (ODE). Numerical simulations are then used to illustrate how variations in key parameters lead to the emergence of network-like spatial structures resembling experimentally observed BMEC patterns. We further propose a data-driven extension of the model that ensures a consistent temporal evolution of the chemo-attractant associated with the observed BMEC dynamics. Finally, we analyze the dynamical behavior of the model in greater detail and introduce a reduced four-dimensional system of 2 × 2 coupled ODEs that captures essential features of the underlying dynamics.

The experimental data used for the mathematical modeling performed here were generated by confocal microscopy of microvasculature composed of BMECs and stem cell derived mural cells, in the Elahi Lab at the Icahn School of Medicine at Mount Sinai. BMECs were isolated from human brain (obtained from ScienCell Research Laboratories, Carlsbad, CA, USA; Cat. No. 1000). Mural Cells were differentiated from iPSC lines according to well-established protocols [14, 15, 16]. Cells were seeded in 96-well plates coated with Geltrex and cultured in Endothelial Cell Medium supplemented with vascular endothelial growth factor (VEGF, 5 ng /mL) and fibroblast growth factor 2 (FGF2, 2 ng/mL).

## 2. On a modified Keller-Segel Equation

### 2.1. Keller-Segel Equations

Relying on observations of *Escherichia coli* placed in an environment containing oxygen and an energy source, Keller and Segel [13] proposed a partial differential equation (PDE) to model the formation of traveling bands in bacterial concentrations observed on plates. The central idea of their work was to interpret this phenomenon as a consequence of chemotaxis: bacteria tend to avoid regions of low concentration and move preferentially toward higher concentrations of the substrate. Additional effects incorporated into the model include random motion (diffusion) and substrate consumption, while growth was not taken into account.

The original Keller–Segel [13] equation is given by

(1) $$\left\{\begin{array}{ll} \frac{\partial b}{\partial t} & =\frac{\partial }{\partial x}(\mu (s)\frac{\partial b}{\partial x})-\frac{\partial }{\partial x}(b\xi (s)\frac{\partial s}{\partial x})\\ \frac{\partial s}{\partial t} & =-k(s)b+D\frac{\partial s}{\partial x^{2}} \end{array}\right.$$

These notations follow those of the original article. Here, b denotes the bacterial concentration and s the substrate concentration. The first term on the right-hand side of the first equation represents bacterial motion in the absence of chemotaxis. The second term on the right-hand side of the first equation describes the chemotactic response of the bacteria. It is assumed that the portion of the bacterial flux resulting from chemotaxis is proportional to the chemical gradient. This assumption is analogous to those used in the derivation of the heat equation.

Since then, numerous variants of the model have been studied both numerically and theoretically. For example, a growth term was introduced and discussed in [17]. We refer to [17, 18, 19, 20] and the references therein for relevant mathematical and numerical studies. The goal of this paper is to propose and analyze a KS-type equation that reproduces relevant patterns for BMEC networks, and to further highlight research directions that appear important for improving our understanding of ND.

### 2.2. On a modified Keller-Segel Equation

We consider the following modified KS type equation,

(2) $$\left\{\begin{array}{ll} u_{t} & =f(u)-b\nabla \cdot (u\nabla v)+d_{u}\Delta u\\ v_{t} & =cu-ev+d_{v}\Delta v \end{array}\right.$$

on a bounded domain Ω with Neuman Boundary Conditions (NBC), and with

$$f\left(u\right)=au\left(1-u\right)\left(u-\gamma \right).$$

The notations are as follows: the term ∇· stands for the divergence operator, the term ∇ for the gradient and the term Δ for the Laplacian. The variable u represents the concentration of endothelial cells and v represents the substrate and the biochemical mechanisms through which cells communicate. This equation is of Keller-Segel type with a modified reaction function. Let us briefly recall the fundamental principles underlying (2). The first equation stipulates that the concentration of cells in an infinitesimal volume will evolve as a result of an intrinsic production function f, the divergence of the concentration of -bu times the gradient of v (which means that if there is more v outside, the concentration at the current point will decrease; this is explained by the divergence theorem), and a diffusion in u, representing the fact that the concentration evolves like the average of its surroundings. As for v, it is produced from u with a rate c and it naturally disappears at a rate e. We introduce the modified function f so that the underlying ODE $u^{\prime }=f(u)$ would have three stationary states: two stable 0 and 1, and one unstable γ. We think that the function f is relevant to model patterns formed by BMEC, because after some time it is observed in experiments that one can roughly divide the two-dimensional well in regions where there is BMEC (u≃1) and regions where there is no BMEC (u≃0). Investigating the dynamical behavior with variation of the parameter γ leads to various configurations, including those observed in experiments. The numerical simulations are presented in section 3. We note that Equation (2) equivalently writes

(3) $$\left\{\begin{array}{ll} u_{t} & =f(u)-b\nabla u\cdot \nabla v-bu\Delta v+d_{u}\Delta u\\ v_{t} & =cu-ev+d_{v}\Delta v \end{array}\right.$$

This last formulation will be used in section 5 for a qualitative description.

### 2.3. Basic Mathematical Analysis of eq. (2)

#### 2.3.1. The underlying ODE

We consider,

(4) $$\left\{\begin{array}{ll} u_{t} & =f(u)\\ v_{t} & =cu-ev \end{array}\right.$$

We have the following result, which follows from straightforward computations,

##### Proposition 1.

Equation (4) admits three stationary points $U_{0}^{*}=(0,\;0)$, $U_{1}^{*}=(\gamma ,\frac{c}{e}\gamma )$, and $U_{2}^{*}=(1,\frac{c}{e})$. $U_{0}^{*}$ and $U_{2}^{*}$ are stable nodes, $U_{1}^{*}$ is a saddle-node. The stable manifold of $U_{1}^{*}$ is given by u=γ. If the initial condition $u_{0}$ satisfies $0\leq u_{0}<\gamma$ then the trajectory converges toward $U_{0}^{*}$, if $u_{0}>\gamma$ the trajectory converges toward $U_{2}^{*}$.

**Figure 1:**
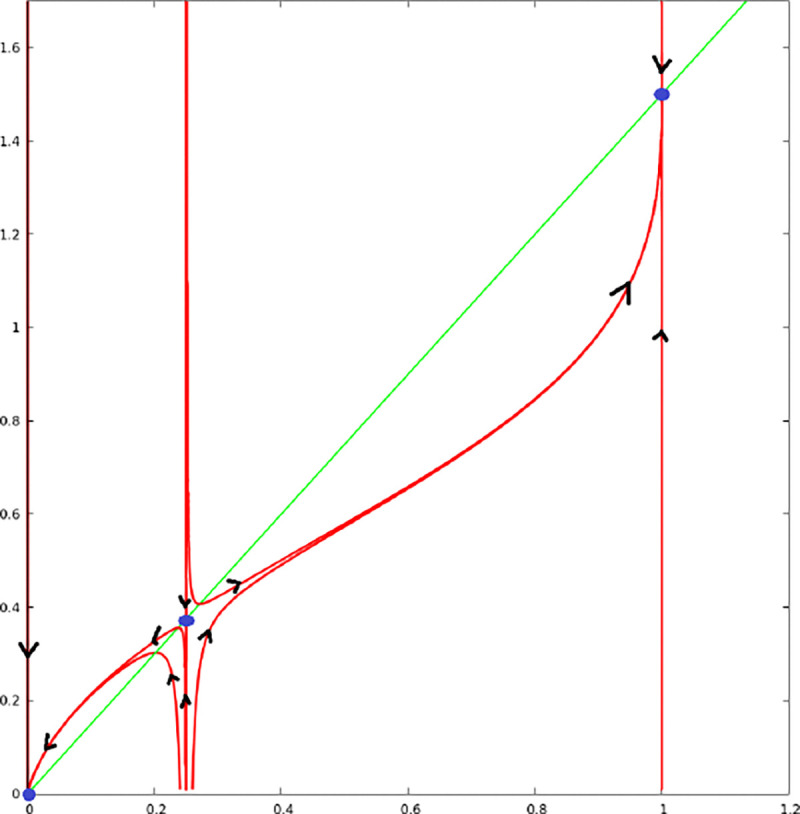
Solutions of the ODE eq. (4)

Figure 1 provides an illustration of the solutions of Equation (4).

#### 2.3.2. Positivity, Mean value

In this section, we provide two basic mathematical results. The first one deals with the positivity of solutions which must be verified in order to satisfy the biological meaning of the variables. The second one deals with the average values.

##### Proposition 2.

The solutions of Equation (2) remain non-negative.

*Proof.* We do not provide here the details of the rigorous proof whose technicality is not in the scope of this article. We refer to [21, 22] for textbooks with technical details. The essential principle at play is that if u or v reaches 0 at an interior point of the domain, then we are at a minimum, the derivative is zero and the Laplacian is non-negative, but the time derivative is non-positive.

The average concentration values are of practical interest since they can serve as a measure of degeneracy for diseased cells. They satisfy specific equations, as the following proposition states. Let

$$\overset{‾}{u}(t)=\int_{\Omega }u(x,t)dx,\;\mathrm{and}\;\overset{‾}{v}(t)=\int_{\Omega }v(x,t)dx,$$

then the following results hold.

##### Proposition 3.

$\overset{‾}{u}$ and $\overset{‾}{v}$ satisfy

(5) $$\left\{\begin{array}{ll} {\overset{‾}{u}}_{t} & =\int_{\Omega }f(u)dx\\ {\overset{‾}{v}}_{t} & =c\overset{‾}{u}-e\overset{‾}{v} \end{array}\right.$$

which for stationary solutions of Equation (2) implies

(6) $$\left\{\begin{array}{ll} 0 & =\int_{\Omega }f(u)dx\\ 0 & =c\overset{‾}{u}-e\overset{‾}{v} \end{array}\right.\;\;4$$

### 3. Numerical Simulations and Emergence of angiogenic patterns

In this section, we describe how numerical simulations of (2) can lead to angiogenic patterns and how variation of the parameter γ affects those patterns. For numerical simulations, we consider a space-discretized version of Equation (2) which corresponds to a finite difference scheme in space (with space step h=1) and a Runge-Kutta resolution method in time.

### 3.1. Parameter setting and Emergence of Angiogenic patterns

The value of parameters are as follows:

(7) $$a=7,b=10,d_{u}=1,c=3,e=2,d_{v}=10.$$

The parameter γ is to be varied between 0 and 1. The model was initially inspired by [17, 20] and subsequently adjusted to capture angiogenic patterns. We choose the initial conditions as follows. The initial concentration of u is distributed over the square domain: it is set to 0.8 in nine small squares that are homogeneously distributed across the spatial domain, and to 0.2 elsewhere. Additionally, a uniformly distributed random perturbation in the range [0,0.2] is added at each point. For v, the initial concentration is set to the constant value 0.5.

The underlying idea is that the concentrations are driven toward 0 or 1 by the reaction function f, in combination with diffusion and transport terms. Choosing γ in the range 0.25–0.29 leads to the formation of angiogenic patterns.

**Figure 2:**
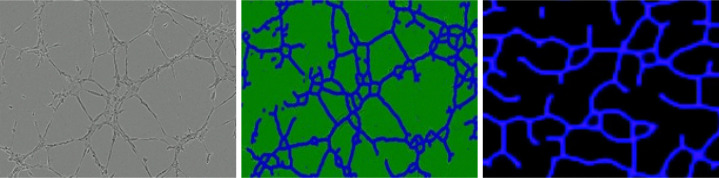
Left: image of BMEC provided by microscopy after 8 hours of evolution. Center: transformed image provided by the free software angiotool. Right: solution of the discretized modified KS model (2) at a fixed time.

Figure 2 illustrates a microscope image of BMEC, along with a corresponding numerical result obtained from Equation (2). The left panel shows the image of the well containing BMEC after 8 hours. The center panel displays the same image after filtering using the AngioTool software [23, 24]. The right panel presents the solution of Equation (2) with γ=0.29 at a fixed time. More details about the simulations are provided in the next paragraph.

### 3.2. Variation of the parameter γ

**Figure 3:**
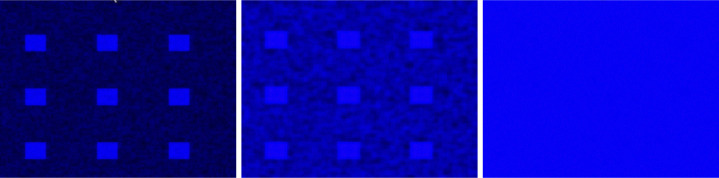
Solution of the modified KS model (2) with γ=0.1 and t∈{0,0.6,2.4}, from left to right. Only u is represented. The solution evolves toward the constant stationary solution $\left(u,v)=(1,\frac{c}{e}\right)$.

**Figure 4:**
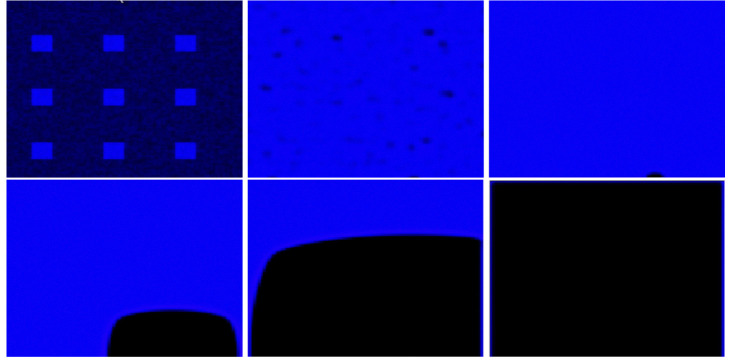
Solution of the modified KS model (2) with γ=0.2 at times t∈0,2,4.6,28.2,67,180, shown from left to right and top to bottom. Only u is represented. After a very short transient period, the solution evolves toward the constant stationary state $\left(u,v)=(1,\frac{c}{e}\right)$ almost everywhere, except for a small region near the bottom-right corner, where it remains at (0, 0). From there, a traveling-wave–type behavior is observed: the solution progressively decreases toward (0, 0), propagating from the bottom-right corner toward the top-left corner. Along the left, top, and right boundaries, the solution remains close to 0.9.

**Figure 5:**
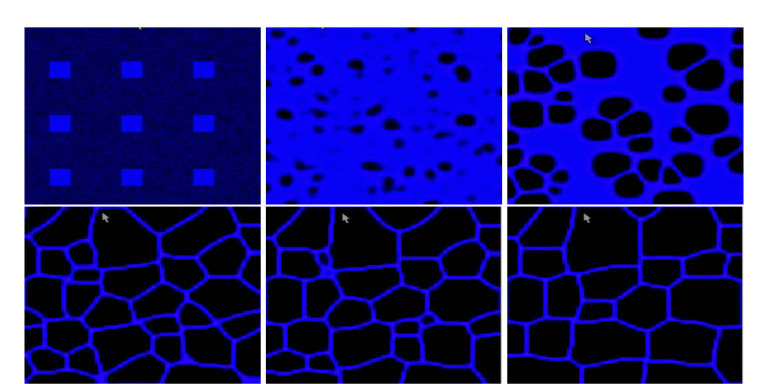
Solution of the modified KS model (2) with γ=0.25 at times t∈0,2.5,7.8,19.2,46.3,180, shown from left to right and top to bottom. Only *u* is represented. The solution initially evolves rapidly toward a state dominated by blue regions (close to the constant stationary solution $\left(u,v)=(1,\frac{c}{e}\right)$), with small patches of black regions (close to (u,v)=(0,0)). From there, the black regions propagate, forming a network-like structure of cells similar to those observed in experiments. The patterns then evolve slowly, with small polygons gradually shrinking and disappearing after a transition through blue. The final state is a network that appears to correspond to a stable minimal-energy configuration, representing the non-homogeneous stationary solution.

**Figure 6:**
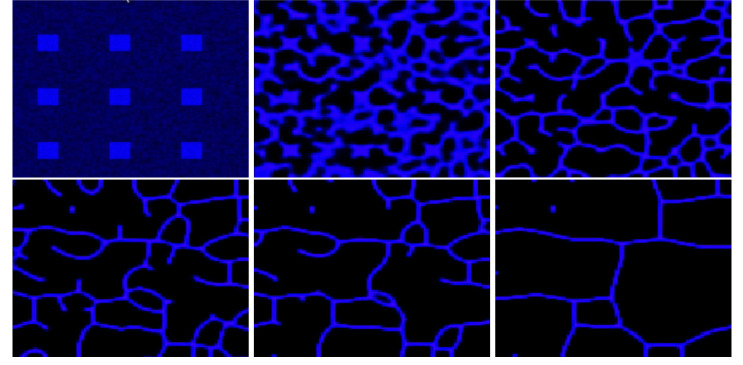
Solution of the modified KS model (2) with γ=0.29 at times t∈0,2.8,6.5,18.5,32.4,180, shown from left to right and top to bottom. Only u is represented. The solution initially evolves rapidly toward a state with both blue and black regions. From there, the black regions gradually expand, forming a cell-like network similar to those observed in experiments. The patterns then evolve slowly, with small polygons shrinking and disappearing, resulting in a degenerated network of cells.

We proceed with the variation of the parameter γ and discuss the variety of observed patterns. If one had to consider only the underlying ODE eq. (4), the principle would be as follows: the smaller the value of γ, the more blue, (i.e., closer to the constant steady state $\left(u,v)=(1,\frac{c}{e}\right)$) the final pattern would be. This is because decreasing γ enlarges the basin of attraction of the steady state u=1 for initial conditions in (0, 1). However, this intuition does not always hold in the full model. We begin with the value γ=0.1, illustrated in Figure 3. For this small value of γ, the solution asymptotically converges to the spatially homogeneous steady state with u=1 and $v=\frac{c}{e}$. Next, we consider γ=0.2, corresponding to Figure 4. The dynamics are very similar to the previous case, at least initially. Interestingly, a small black region (i.e. close to the steady state (u,v)=(0,0)) persists in the lower right corner of the domain, leading to a traveling wave solution. Asymptotically, the domain becomes almost entirely black, except for thin regions near the boundary. Simulations for γ=0.25 are shown in Figure 5. In this case, the solution initially evolves rapidly toward a state dominated by blue, with small regions of black. Subsequently, the black regions propagate, leading to structures similar to those observed in experiments. The patterns then evolve more slowly, with small polygons shrinking and eventually disappearing after transitioning through a blue phase. The final state is a network that suggests a stable, minimal-energy configuration, corresponding to a spatially non-homogeneous steady-state solution. This description indicates a pronounced temporal structure with distinct dynamical phases. We will discuss this point in more detail in section section 5 by examining the time evolution at specific spatial positions. Finally, for γ=0.29 (see Figure 6), the solution initially evolves rapidly toward a state with a mixture of blue and black regions. From there, the area occupied by black regions tends to increase, leading to a structure similar to those observed in experiments. Subsequently, as before, the patterns evolve slowly, with small polygons gradually shrinking and disappearing. The resulting stationary pattern resembles a degenerate network of cells.

## 4. Data Driven reproduction of Chemo-attractant evolution

**Figure 8:**
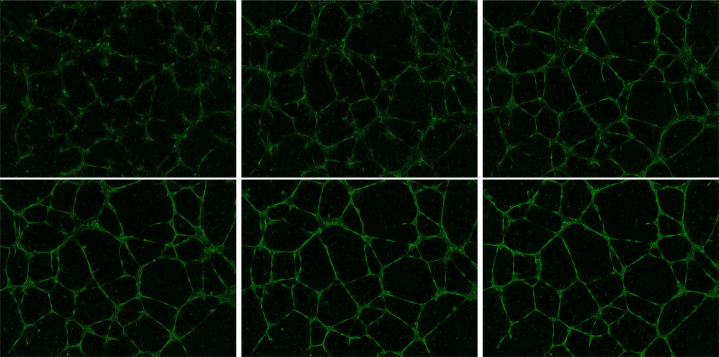
Original images of BMEC obtained by microscopy at the Elahi Lab at times t=2,4,6,8,10 and 12 hours.

In this section, we describe a procedure to reconstruct the temporal evolution of the cell patterns by combining elements of Eq. (2) with experimentally observed data. To this end, fifteen microscopy images were used to generate the reconstruction; six representative images are shown in Fig. 8. The resulting movie illustrating the evolution of the cellular patterns is provided as supplementary material accompanying the manuscript.

Our objective is to find the value of fields v that correspond to the temporal evolution of the cellular patterns. To this end, we treat two consecutive microscopy images, denoted by u(t) and u(t+1), as given data and compute the corresponding field v by solving the equation

(8) $$u(t+1)-u(t)=f(u(t))-b\nabla \cdot (u(t)\nabla v(t))+d_{u}\Delta u(t)$$

or equivalently

(9) $$-\nabla u(t)\cdot \nabla v(t)-u\Delta v(t)=\frac{1}{b}\left(u(t+1)-u(t)-f(u(t))-d_{u}\Delta u(t)\right)$$

**Figure 9:**
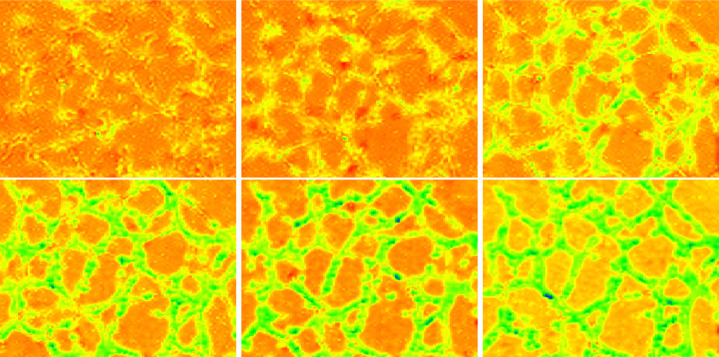
The six fields v obtained by solving equation (9) at t=2,4,6,8,10 and 12 hours.

Equation (9) is an elliptic partial differential equation for v. Since u>0, classical elliptic theory ensures the existence and uniqueness of the solution up to an additive constant. However, solving this equation numerically remains challenging. To address this issue, we adapted the GMRES (Generalized Minimal Residual) method, an iterative Krylov subspace algorithm that computes approximate solutions by minimizing the residual norm at each iteration [25]. The details of the numerical procedure are provided in the Appendix, and the corresponding C++ implementation is available. The six obtained fields at t=2,4,6,8,10 and 12 are illustrated in figure 9 .

## 5. Numerical Simulations: a three time-phase structure for γ=0.25

**Figure 10:**
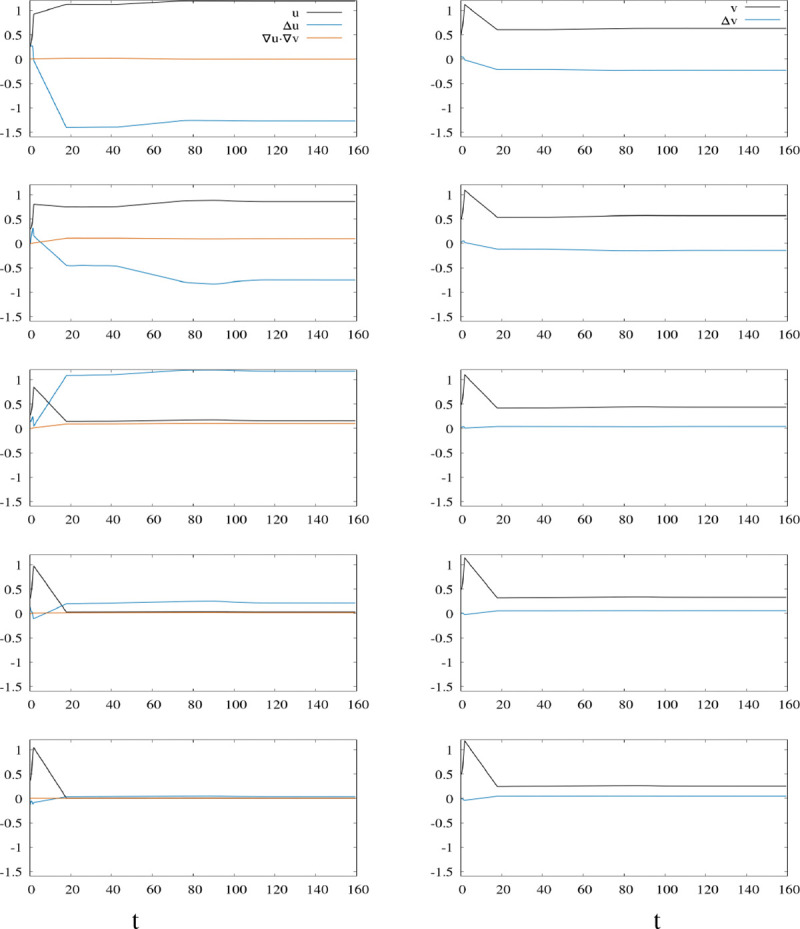
This figure illustrates the time evolution of the solution of the discretized version of the modified KS model (2) with γ=0.25 at several consecutive fixed spatial positions. The panels on the left show the time evolution of u (black), Δu (blue), and ∇u⋅∇v (orange), while the panels on the right display the evolution of v (black) and Δv (blue). A clear temporal structure emerges, particularly in the time course of v, with slopes indicating three distinct time scales that may be classified as very fast, fast, and slow. The first row corresponds to the position (x,y)=(50,50). Comparison with the other rows shows that u and v asymptotically approach a local maximum stationary value, while Δu and Δv converge to a negative stationary value. The second row corresponds to (x,y)=(51,50), where u and v reach a relatively high stationary value compared to neighboring positions, as confirmed by the negative asymptotic values of Δu and Δv. A similar qualitative behavior, albeit with different asymptotic states (e.g., minima instead of maxima), is observed at (x,y)=(52,50) and (x,y)=(53,50).

In this section, we further proceed with a more in-depth qualitative analysis of the numerical solutions for γ=0.25. Our goal is to investigate what are the dynamical mechanisms that lead to the patterns observed in the previous section for this particular value. Since in the setting considered here, the stationary patterns appear visually as networks of a blue color in a black background, we focus on how solutions evolve in time, at some fixed spatial close positions, where u is asymptotically close to its maximum value (close to the value one which appears in blue) or close to its minimum value (close to zero which appears in black). Interestingly, when looking at the time evolution at these fixed positions, we can distinguish three phases in time. This seems to correspond to dynamics at different time scales: very fast, fast and slow. Biological processes with different time scales have been widely observed and reported, specifically in neuroscience context. It has also been studied theoretically. We refer for example to the textbook [26] and the numerous references cited in there; the topic is commonly referred as slow-fast dynamics in the field of dynamical systems and computational neuroscience. A detailed investigation of the theoretical slow-fast structure of the system under consideration is not in the scope of this article. Figure 10 illustrates the time evolution of relevant quantities: in the left panels, we represent the time evolution of u, Δu and ∇u⋅∇v, while in the right panels, we illustrate the time evolution of v and Δv. This allows us to get a glimpse about the factors that drive the dynamics locally in space. To highlight the mechanisms at play, we describe hereafter in some detail two opposite dynamical behaviors (at fixed space positions) for which u is asymptotically close to its maximum and minimum values (see Figure 10, first and third row). Analog arguments hold for dynamics at other positions. The first row of Figure 10 illustrates the dynamics of the above mentioned quantities at the space point (50, 50) for which u is asymptotically at a local maximum. We can distinguish three phases. During the first phase, u increases rapidly. Analog behavior is observed for v. The term ∇u⋅∇v is close to zero ( *i*.*e*. ∇u and ∇v are almost orthogonal) and negligible. The term Δu is positive. This contributes to the fast increase in u. We note however that Δu decreases with time. This means that at this point, u is smaller than the average values of its neighbors, but tends to increase faster than its neighbors. Meanwhile, analogously, Δv is positive and decreases. At some point in time, we observe a sudden change in the time derivative of u. We associate this sudden change with the start of the second phase. After that, the variable u is still increasing, but in a slower fashion, meanwhile, Δu becomes negative which contributes to slowing down u. The sudden change in the evolution of the variable v is even more spectacular. The second phase starts indeed with a sudden change in the time derivative of v from positive to negative: the variable v starts to decrease abruptly during the second phase. Δv becomes negative and decreases. Negative values of Δv contribute to the increase of u and the decrease of v. Note that b and $d_{v}$ have large values which intensifies the impact of Δv values. At some point, we observe again an abrupt change in the time derivative for quantities u, v, Δu and Δv: the time derivative becomes close to zero and we associate this with the beginning of the third phase. After that, all the quantities remain almost steady with a notable small increase in u and Δu. We note that u and v reach stationary positive values, which are local maximums. Accordingly, Δu and Δv reach stationary negative values. We note that at the equilibrium, for the first equation u>1 which means that f(u)<0, Δu<0 (negative contributions), -buΔv>0 (positive contribution). Here is an effect of the chemotaxis term: a local maximal concentration in v contributes to a local maximal concentration in u, and compensates the negative contributions of the growing term (here shrinking) f(u) and the diffusive term Δu. The third row of Figure 10 illustrates the dynamics at the spatial point (x,y)=(52,50). During the first phase, as described for (x,y)=(50,50), u and v increase rapidly. The term ∇u⋅∇v increases slowly. The term Δu has a small fast increase followed by a small fast decrease. This interestingly tells us about the growing dynamics of u in comparison to its neighbors. Since Δu>0 during this phase, the quantity u is lower than the average value of its neighbors; when Δu increases it tells us that the neighbors are growing faster and when Δu decreases it tells us that the quantity u is growing faster at this space point than at the neighboring positions in average. At the end of phase 1, it has a small positive value. We note that since u∈(γ,1), the growth factor f(u) has an increasing effect. During phase 1, Δv has a small increase followed by a small decrease and reaches a value close to zero. In this case, we observe that the increase in u is caused by the growth and diffusive factors. The chemotaxis terms have a small negative effect. During phase 2, u and v have a fast decrease. This contrasts with the observed behavior at (x,y)=(50,50). ∇u⋅∇v continues to increase at a slow constant rate. Δu increases rapidly and reaches a high positive value. Δv has a small slow increase. From this observation, we deduce that ∇u⋅∇v and Δv, *i*.*e* the chemotaxis terms, are the factors that first contribute to the decrease of u. Even though, they seem to be small it is sufficient because of the large values of b and $d_{v}$. At some point, u crosses γ downwards which means that f(u) starts also to contribute to the decrease in u. During the last phase, v, Δv and ∇u⋅∇v remain constant. There are some slight evolutions for u and Δu, but at some point all quantities reach a stationary state. By contrast with the observation made at (x,y)=(50,50), we note that at the equilibrium, for the first equation, u reach a quite low value with u∈(0,γ), which means that f(u)<0. We note also that -buΔv<0,-b∇u⋅∇v<0. All there are negative contributions. The term Δu>0 contributes positively. We briefly mention the dynamics at the spatial point (54, 50) at which u is at its local minimum, see the last row of Figure 10. Here, the dynamics are close to that described at (x,y)=(52,50). The main difference being that u is almost equal to 0 and Δu is much smaller. We have also that ∇u⋅∇v is almost 0. At the end, we observe that u(50,50)>u(51,50)>u(52,50)>u(53,50)>u(54,50).

## 6. On a 2 × 2 analog coupled model

With the goal to investigate some mathematical mechanisms at play for a simpler and more tractable model, we propose the following ODE,

(10) $$\left\{\begin{array}{ll} u_{1t}= & f\left(u_{1}\right)-bu_{1}\left(v_{2}-v_{1}\right)+d_{u}\left(u_{2}-u_{1}\right)\\ v_{1t}= & cu_{1}-ev_{1}+d_{v}\left(v_{2}-v_{1}\right)\\ u_{2t}= & f\left(u_{2}\right)-bu_{2}\left(v_{1}-v_{2}\right)+d_{u}\left(u_{1}-u_{2}\right)\\ v_{2t}= & cu_{2}-ev_{2}+d_{v}\left(v_{1}-v_{2}\right) \end{array}\right.$$

The value of parameters are as follows:

(11) $$a=7,d_{u}=1,c=3,e=2,d_{v}=10,\gamma =0.25,b\in [10,\;25]$$

Equation (10) is a two×two coupled ODE which mimics Equation (3) in the following manner: the nonlinear factor (growth factor) is the same, the diffusive terms appear for example in $u_{2}-u_{1}$, and the term $u_{1}(v_{2}-v_{1})$ is inspired from u∇v. For certain values of the parameters, Equation (10) admits four stationary locally asymptotically stable solutions coexisting: $U_{1}^{*}=(0,\;0,\;0,\;0)$, $U_{3}^{*}=(1,\frac{c}{e},1,\frac{c}{e})$, $U_{8}^{*}=(u_{1}^{8*},v_{1}^{8*},u_{2}^{8*},v_{2}^{8*})$, $U_{9}^{*}=(u_{1}^{9*},v_{1}^{9*},u_{2}^{9*},v_{2}^{9*})$ with $u_{1}^{9*}=u_{2}^{8*}$ and $\gamma <u_{2}^{8*}<1<u_{1}^{8*}$. Therefore, Equation (10) admits stationary solutions that represent two high concentration of cells, no cells, and coexistence of high and low concentration of cells. We note that there is symmetry in the system: if $\left(u_{1},v_{1},u_{2},v_{2}\right)(t)$ is a solution, so is $\left({\tilde{u}}_{1},{\tilde{v}}_{1},{\tilde{u}}_{2},{\tilde{v}}_{2}\right)(t)=\left(u_{2},v_{2},u_{1},v_{1}\right)(t)$. We start with the following proposition that ensures that variables remain positive.

### Proposition 4.

The set $G=\left\{u_{1}>0,v_{1}>0,u_{2}>0,v_{2}>0\right\}$ is positively invariant for Equation (10).

*Proof.* We note that the set $u_{1}=u_{2}=0$ is an invariant manifold. Next, we observe that $u_{1}^{\prime }>0$ *at* $u_{1}=0$ *and* $u_{2}>0$. The same argument holds for the other variables.

The next proposition characterizes the stationary solution.

### Proposition 5.

The stationary solutions of Equation (10) satisfy the following equations.

(12) $$\left\{\begin{array}{ll} u_{2}= & \phi \left(u_{1}\right)\\ u_{1}= & \phi \left(u_{2}\right)\\ v_{1}= & \alpha_{1}u_{1}+\alpha_{2}u_{2}\\ v_{2}= & \alpha_{1}u_{2}+\alpha_{2}u_{1} \end{array}\right.$$

with

(13) $$\phi \left(u_{1}\right)=u_{1}+\frac{f\left(u_{1}\right)}{bu_{1}\left(\alpha_{1}\;-\;\alpha_{2}\right)\;-\;d_{u}}$$

and

(14) $$\alpha_{1}=\frac{c\left(d_{v}\;+\;e\right)}{{\left(d_{v}\;+\;e\right)}^{2}\;-\;d_{v}^{2}},\alpha_{2}=\frac{c}{d_{v}({\left(1\;+\;\frac{e}{d_{v}}\right)}^{2}\;-\;1)}.$$

Equivalently, stationary solutions of Equation (10) *satisfy the fixed point equation*

(15) $$u_{1}=\phi \circ \phi \left(u_{1}\right)$$

*along with $v_{1}$*, $u_{2}$, $v_{2}$ *solutions of* Equation (12).

#### Dynamical description

**Figure 11:**
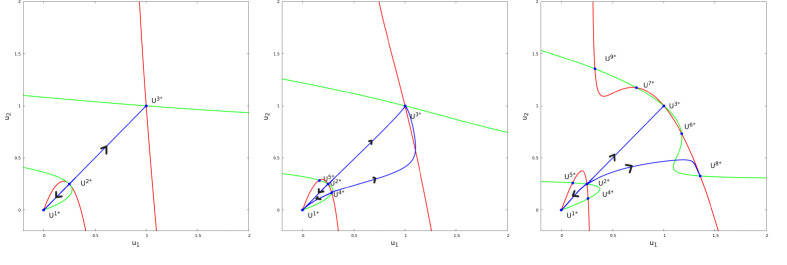
Stationary solutions and some trajectories of Equation (10) projected in the $u_{1}$, $u_{2}$ phase plan. Left panel, b=10, middle panel, b=15, right panel, b=25. The red curves represent $u_{2}=\phi \left(u_{1}\right)$. Green curves represent $u_{1}=\phi \left(u_{2}\right)$. Blue dots are projections of stationary solutions at the intersection of red and green curves. As the parameter b increases from 10 to 25, new stationary points appear. On the left side, for b=10, we observe only the three stationary solutions where $u_{1}=u_{2}$ and $v_{1}=v_{2}$ which are known from the study of Equation (4). At b=15, we observe two unstable stationary solutions $U^{4*}$ and $U^{5*}$. At b=25, we observe four more stationary solutions $U^{6*}$, $U^{7*}$, which are unstable and $U^{8*}$, $U^{9*}$, which are locally asymptotically stable. Blue curves represent the projections of some trajectories of Equation (10) projected in the $u_{1}$, $u_{2}$ phase plan. Note that there is a symmetry in the system.

**Figure 12:**
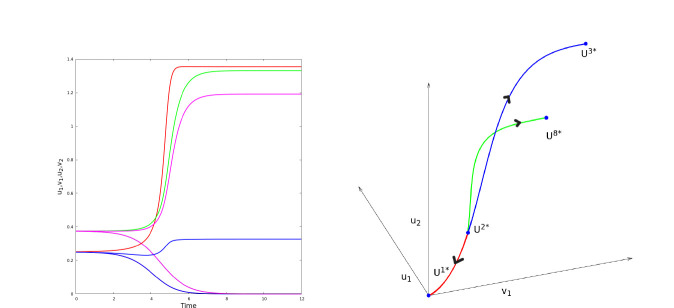
Solutions of Equation (10) for b=25. The left panel illustrates $u_{1}$ (red), $v_{1}$ (green), $u_{2}$ (blue), $v_{2}$ (magenta) as functions of time, for two initial conditions close to the stationary point $U^{2*}=\left(\gamma ,\frac{c}{e}\gamma ,\gamma ,\frac{c}{e}\gamma \right)$. We observe that one of the solutions converges toward $U^{8*}=\left(u_{1}^{8*},v_{1}^{8*},u_{2}^{8*},v_{2}^{8*}\right)$ (with $\gamma <u_{2}^{8*}<1<u_{1}^{8*}$) while the other one converges toward $U^{1*}=(0,\;0,\;0,\;0)$. The right panel illustrates three heteroclinic orbits going from $U^{2*}$ towad $U^{1*}$ (red), $U^{3*}$ (blue) and $U^{8*}$ (green) as t goes from -∞ to +∞.

For parameters as in Equation (11), after numerical investigation, the dynamics can be summarized as follows: for b close to 10, there is only three stationary points $U^{1*}=(0,\;0,\;0,\;0)$, $U^{2*}=(\gamma ,\frac{c}{e}\gamma ,\gamma ,\frac{c}{e}\gamma )$ and $U^{3*}=(1,\frac{c}{e},1,\frac{c}{e})$. $U^{1*}$ and $U^{3*}$ are locally asymptotically stable, $U^{2*}$ is unstable. As b increases, two new unstable stationary points (with $u_{1}\neq u_{2}$), $U^{4*}$, $U^{5*}$ appear. As b increases more, four new stationary points appear: two unstable $U^{6*}$, $U^{7*}$ and two locally asymptotically stable $U^{8*}$, $U^{9*}$. We refer to Figure 11 and Figure 12 for illustrations. Finally, we note that stationary solutions $U^{8*}$, $U^{9*}$ provide illustrations for the effects of nonzero growth factors, diffusive factors and chemotactic factors at equilibrium. Indeed, at $U^{8*}$, we have a negative growth factor $f(u_{1}^{8*})<0$ and a negative diffusive factor $u_{2}^{8*}-u_{1}^{8*}<0$ (local maximum, negative diffusion), compensated by a positive chemotactic factor $-bu_{1}(v_{2}-v_{1})>0$; a positive growth factor $f(u_{2}^{8*})>0$ and a positive diffusive factor $u_{1}^{8*}-u_{2}^{8*}<0$ (local minimum, positive diffusion), compensated by a negative chemotactic factor $-bu_{2}(v_{1}-v_{2})<0$. We recover in this low dimensional system the principles that led to the formation of BMEC patterns in the KS PDE. It follows that Equation (10) represents a low-dimensional prototype for non homogeneous concentrations states featuring growth, diffusive and chemotactic factors.

## 7. Conclusion

In this article, we have introduced a modified KS model that reproduces the patterns observed in *in vitro* BMEC growth experiments and provided mathematical insights into the mechanisms driving this pattern formation. Based on this model, we have also computed distributions of chemoattractant concentrations that are consistent with the time evolution of the experimental growth patterns. New data are currently being generated, and our dynamical-systems approach will be applied in combination with statistical and machine learning methods, with the goal of reducing the dimensionality required for characterization and extracting meaningful biological parameters. This research is part of a broader effort that includes developing biochemical mathematical models for angiogenesis, *in vitro* models incorporating BMECs, astrocytes, and neurons, as well as three-dimensional models of blood flow, ultimately aiming to create in silico platforms to support the development of new therapeutics for neurodegenerative diseases.

## Figures and Tables

**Figure 7:**
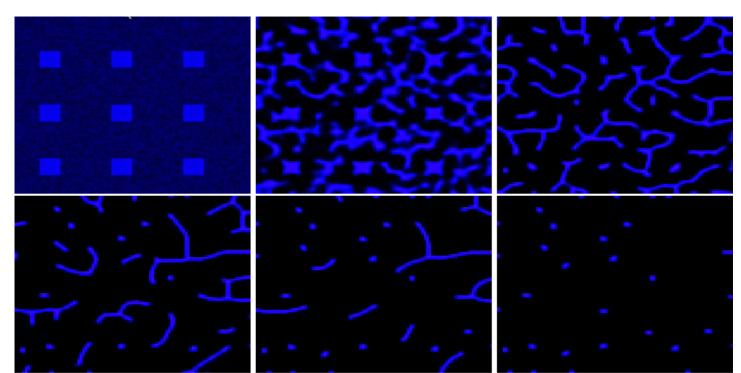
Solution of the modified KS model (2) at six fixed times spanning t=0 to 180, shown from left to right and top to bottom. Only u is represented. The solution initially evolves rapidly toward a state with both blue and black regions. In this case, no polygons form. At the final stage, a few blue spots remain on a predominantly black background.

## Appendix A. Implementation of the GMRES method for solving the elliptic equation for the field v

In this study, we were led to the problem of computing a numerical solution to a linear elliptic PDE of the form:

$$-\nabla u(t)\cdot \nabla v(t)-u\Delta v(t)=\frac{1}{b}(u(t+1)-u(t)-f(u(t))-d_{u}\Delta u(t))$$

with NBC on ∂Ω. Here, u is known (this is the data) and the unknown is v. Discretization of the operator -∇u(t)⋅∇v(t)-uΔv(t) using finite differences leads to a sparse linear system:

(A.1)
AX=B,

where for our choice of space step discretization $A\in R^{10^{8}}$. This is numerically challenging. The *Generalized Minimal Residual (GMRES)* method [25] is an iterative method which does not require to explicit A, rather, it only requires to compute AX which belongs to $R^{10^{4}}$. This was indeed very efficient to solve our problem. GMRES is an iterative Krylov subspace method, which constructs an approximate solution $X_{k}$ in the Krylov subspace

(A.2)
$${𝒦}_{k}\left(A,R_{0}\right)=span\left\{R_{0},AR_{0},A^{2}R_{0},\ldots ,A^{k-1}R_{0}\right\},$$

where $R_{0}=B-AX_{0}$ is the initial residual. At each iteration, GMRES computes

(A.3)
$$r_{k}=\underset{X\in X_{0}+{𝒦}_{k}}{min}{\left\|B-AX\right\|}_{2}.$$

This constitutes the main loop of the algorithm that we implemented, which stops when $r_{k}$ reaches a small threshold value ϵ. We developed our own C++ code (supplementary material). For our problem, we set ϵ=0.1. At each step, the algorithm defines a new vector $V_{k}$, such that the sequence $V_{1},\ldots .,V_{k}$, is orthonormal. This goes as follows, initiate

$$V_{1}=\frac{r_{0}}{\left\|r_{0}\right\|.}$$

Assuming $V_{1},\ldots ,V_{k-1}$ are known and orthonormal, then we seek

$${\tilde{V}}_{k}=AV_{k-1}-\sum_{j=1}^{k-1}\beta_{j}V_{j}.$$

Orthogonality condition

$$\left({\tilde{V}}_{k},V_{j}\right)=0$$

leads to

$$\beta_{j}=\left(AV_{k-1},V_{j}\right),j\in \left\{1,\ldots ,k-1\right\}.$$

Then we normalize

$$V_{k}=\frac{{\tilde{V}}_{k}}{\left\|{\tilde{V}}_{k}\right\|}.$$

This is used to compute $r_{k}$ as

$$r_{k}=\underset{X_{k}=X_{0}+\sum_{i=1}^{k}y_{i}V_{i}}{min}{\left\|B-AX_{k}\right\|}_{2}.$$

However, we remark that

$$B-AX_{k}=R_{0}-\sum_{i=1}^{k}y_{i}AV_{i}$$

and that

$$AV_{i}-\sum_{j=1}^{i}\left(AV_{j},V_{i}\right)V_{j}={\tilde{V}}_{i+1}=\left\|{\tilde{V}}_{i+1}\right\|V_{i+1}$$

which gives

$$r_{k}=\underset{y_{1},y_{2},\ldots ,y_{k}}{min}{\left\|R_{0}-\sum_{i=1}^{k}y_{i}(\sum_{j=1}^{i+1}h_{ji}V_{j})\right\|}_{2}$$

where

$$h_{ji}=\left(AV_{j},V_{i}\right)1\leq j\leq i,h_{i+1,i}=\left\|{\tilde{V}}_{i+1}\right\|.$$

This rewrites in matricial form

$$r_{k}=\underset{y\in R^{k}}{min}\left\|R_{0}-(VH)y\right\|$$

where

$$V=\left(V_{1},\ldots ,V_{k+1}\right)$$

and

$$H=\left(\begin{array}{cccc} h_{11} & h_{12} & \cdots & h_{1k}\\ h_{21} & h_{22} & \cdots & h_{2k}\\ 0 & h_{32} & \cdots & h_{3k}\\ \vdots & \vdots & \ddots & \vdots \\ 0 & 0 & \cdots & h_{k,k}\\ 0 & 0 & \cdots & h_{k+1,k} \end{array}\right).$$

Since V is orthonormal and $R_{0}=V_{1}r_{0}$, multiplying by V gives

$$r_{k}=\underset{y\in R^{k}}{min}\left\|r_{0}e_{1}-Hy\right\|.$$

Then we further write

$$r_{k}=\underset{y\in R^{k}}{min}\left\|r_{0}Qe_{1}-QHy\right\|,$$

with

$$Q=G_{k}G_{k-1}\ldots G_{1}$$

and

$$G_{i}=\left(\begin{array}{cccccc} 1 & & & & & \\ & \ddots & & & & \\ & & 1 & & & \\ & & & \begin{array}{c} cos\;\theta \\ sin\;\theta \end{array} & \begin{array}{c} -sin\;\theta \\ cos\;\theta \end{array} & \\ & & & & & 1\\ & & & & & \ddots \end{array}\right)$$

where θ is chosen so that the (i+1,i) entry of $G_{i}\ldots G_{1}H$ is zero. It follows that QH is an upper triangular matrix with k+1 rows and k columns, with (k+1)-th row consisting entirely of zeros. From this, we deduce that $r_{k}={\left(r_{0}Qe_{1}\right)}_{k+1}$, and thus no explicit computation of y is required until the while loop terminates. Finally, we summarize the algorithm below.

**Algorithm 1:** Generalized Minimal Residual Method (GMRES) to solve the elliptic euqtion

1. Choose an initial guess $X_{0}\in R^{n}$ and a tolerance ε>0.
2. Compute the initial residual
$R_{0}=B-AX_{0},\;r_{0}={\left\|R_{0}\right\|}_{2}.$
3. If $r_{0}<\varepsilon$, stop and set $X=X_{0}$.
4. Set
$V_{1}=\frac{R_{0}}{{\left\|R_{0}\right\|}_{2}}$
5. Enter the main while loop, while $r_{k}>\varepsilon$ do:
(a) Compute
$h_{i,k},V_{k+1},1\leq i\leq k+1$
as discussed above
(b) Apply the previously computed matrix rotations
$G_{1},G_{2},\ldots ,G_{k-1}$
to the vector
${\left(h_{1,k},h_{2,k},\ldots ,h_{k+1,k}\right)}^{T}.$
(c) Compute a new matrix rotation $G_{k}$ such that
$G_{k}\left[\begin{array}{c} h_{k,k}\\ h_{k+1,k} \end{array}\right]=\left[\begin{array}{c} \sqrt{h_{k,k}^{2},+h_{k+1,k}^{2}}\\ 0 \end{array}\right].$
(d) Apply $G_{k}$ and compute the residual norm $r_{k}$.
(e) If $r_{k}\leq \varepsilon$, stop.
6. Solve the upper triangular system in $R^{k}$
$Ry=r_{0}Qe_{1},$
7. Compute the approximation of v,
$X_{k}=X_{0}+Vy.$
